# 2,3-*O*-(*S*)-Benzyl­idene-2-*C*-methyl-d-ribono-1,4-lactone

**DOI:** 10.1107/S1600536809032796

**Published:** 2009-08-22

**Authors:** K. Victoria Booth, Sarah F. Jenkinson, George W. J. Fleet, David J. Watkin

**Affiliations:** a Department of Organic Chemistry, Chemistry Research Laboratory, Department of Chemistry, University of Oxford, Oxford, OX1 3TA, UK; b Department of Chemical Crystallography, Chemistry Research Laboratory, Department of Chemistry, University of Oxford, Oxford, OX1 3TA, UK

## Abstract

The crystal structure of the title compound, C_13_H_14_O_5_, establishes (i) the (*S*) – rather than (*R*) – configuration at the acetal carbon and (ii) that both the acetal and the lactone form five- rather than six-membered rings; the absolute configuration is determined by the use of 2-*C*-methyl-d-ribono-1,4-lactone as the starting material. The compound consists of hydrogen-bonded chains of mol­ecules running along the *a* axis; there are no unusual packing features. Only classical hydrogen bonding has been considered.

## Related literature

For the synthesis of sugar lactones and their use as building blocks, see: Lundt & Madsen (2001 ▶); Hotchkiss, Soengas *et al.* (2007 ▶); Booth *et al.* (2008 ▶, 2009 ▶); Jenkinson *et al.* (2007 ▶); Hotchkiss, Kato *et al.* (2007 ▶); Chen & Joullie (1984 ▶); Dho *et al.* (1986 ▶); Baird *et al.* (1987 ▶). For the structures of benzyl­idene acetals, see: Baggett *et al.* (1985 ▶); Zinner *et al.* (1968 ▶).
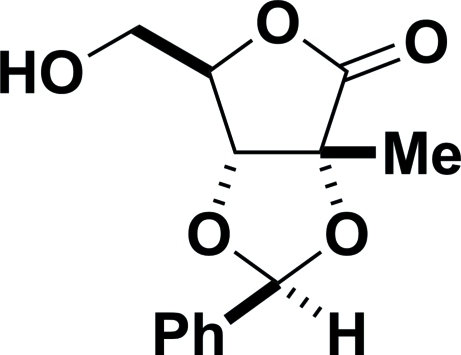


## Experimental

### 

#### Crystal data

C_13_H_14_O_5_
*M* *_r_* = 250.25
Orthorhombic, 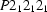

*a* = 8.6170 (2) Å
*b* = 10.4615 (3) Å
*c* = 13.2693 (5) Å
*V* = 1196.18 (6) Å^3^
*Z* = 4
Mo *K*α radiation
μ = 0.11 mm^−1^
*T* = 150 K
0.50 × 0.40 × 0.40 mm

#### Data collection

Nonius KappaCCD diffractometer
Absorption correction: multi-scan (*DENZO*/*SCALEPACK*; Otwinowski & Minor, 1997 ▶) *T*_min_ = 0.91, *T*_max_ = 0.96
8306 measured reflections
1547 independent reflections
1369 reflections with *I* > 2.0σ(*I*)
*R*_int_ = 0.036

#### Refinement

*R*[*F* ^2^ > 2σ(*F* ^2^)] = 0.033
*wR*(*F* ^2^) = 0.075
*S* = 0.96
1547 reflections
163 parameters
H-atom parameters constrained
Δρ_max_ = 0.21 e Å^−3^
Δρ_min_ = −0.18 e Å^−3^

### 

Data collection: *COLLECT* (Nonius, 1997-2001 ▶).; cell refinement: *DENZO*/*SCALEPACK* (Otwinowski & Minor, 1997 ▶); data reduction: *DENZO*/*SCALEPACK*; program(s) used to solve structure: *SIR92* (Altomare *et al.*, 1994 ▶); program(s) used to refine structure: *CRYSTALS* (Betteridge *et al.*, 2003 ▶); molecular graphics: *CAMERON* (Watkin *et al.*, 1996 ▶); software used to prepare material for publication: *CRYSTALS*.

## Supplementary Material

Crystal structure: contains datablocks global, I. DOI: 10.1107/S1600536809032796/lh2882sup1.cif

Structure factors: contains datablocks I. DOI: 10.1107/S1600536809032796/lh2882Isup2.hkl

Additional supplementary materials:  crystallographic information; 3D view; checkCIF report

## Figures and Tables

**Table 1:** Hydrogen-bond geometry (Å, °)

*D*—H⋯*A*	*D*—H	H⋯*A*	*D*⋯*A*	*D*—H⋯*A*
O18—H181⋯O9^i^	0.84	2.02	2.801 (3)	153

Symmetry code: (i) 
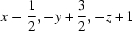
.

**Fig. 1.**
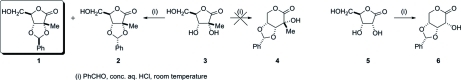
Synthetic Scheme

**Fig. 2.**
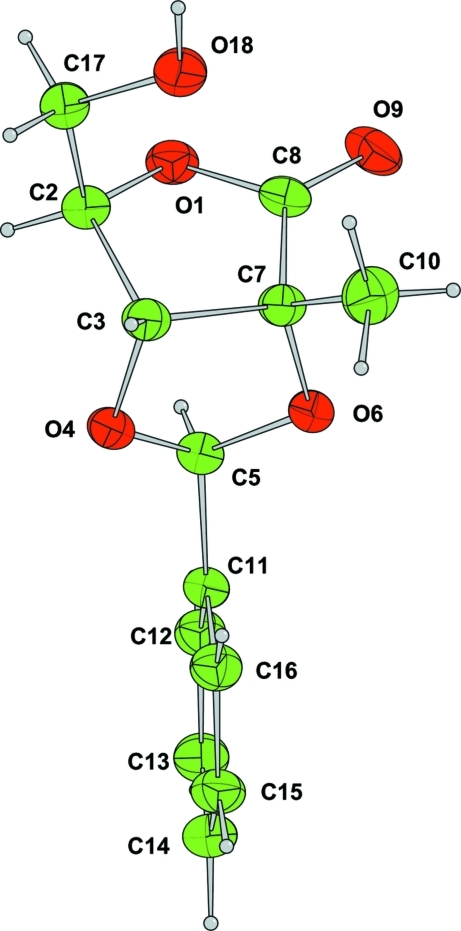
The title compound with displacement ellipsoids drawn at the 50% probability level. H atoms are shown as spheres of arbitary radius.

**Fig. 3.**
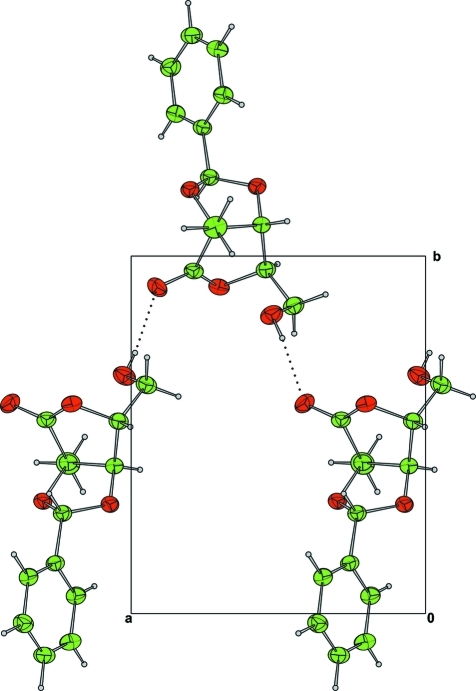
Packing diagram for the title compound projected along the c-axis. Hydrogen bonds are indicated by dotted lines.

## supplementary crystallographic information

### Comment

Lactones have been widely used for the enantiospecific synthesis of complex chiral targets (Lundt & Madsen, 2001). 2-*C*-Methyl-D-ribono-1,4-lactone 3 (Fig. 1) has recently become a readily available starting material (Hotchkiss, Soengas *et al.*, 2007; Booth *et al.*, 2008) and has been used in the synthesis of doubly branched sugars (Booth *et al.*, 2009), 2-*C*-methyl nucleosides (Jenkinson *et al.*, 2007) and complex piperidines (Hotchkiss, Kato *et al.*, 2007). D-Ribono-1,4-lactone 5 with benzaldehyde and concentrated aqueous hydrochloric acid forms a 5 ring benzylidene acetal - 6-ring lactone 6 (Fig. 1). The structure of 6 was established by X-ray crystallographic analysis (Baggett *et al.*, 1985) which corrected the original erroneous 6 ring benzylidene acetal - 5-ring lactone structure proposed (Zinner *et al.*, 1968). The protected 1,5-lactone 6 leaves only the C-2 OH unprotected and has been widely used as a chiron (Chen & Joullie, 1984; Dho *et al.*, 1986; Baird *et al.*, 1987). It was hoped that the analogous reaction with 2-*C*-methyl lactone 3 would form the analogous lactone 4; however, treatment of 3 with benzaldehyde and concentrated aqueous hydrochloric acid gave as the major product a mixture of epimeric 1,4-lactones 1 and 2; although it was not possible to separate 1 and 2 by chromatography, suitable crystals of the major component 1 were obtained and the structure of a 5 ring benzylidene acetal - 5-ring lactone, together with the (*S*) stereochemistry at the acetal carbon, was firmly established (Fig. 2).

The compound consists of H—O···H hydrogen bonded chains of molecules running along the *a*-axis (Fig. 3); there are no unusual packing features. Only classical hydrogen bonding has been considered.

### Experimental

The title compound was recrystallized from a mixture of diethyl ether and petrol by slow evaporation: m.p. 369–372 K; [α]_D_^18^ -38.7 (*c*, 0.86 in CHCl_3_).

### Refinement

In the absence of significant anomalous scattering, Friedel pairs were merged.

The H atoms were all located in a difference map, but those attached to carbon atoms were repositioned geometrically. The H atoms were initially refined with soft restraints on the bond lengths and angles to regularize their geometry (C—H in the range 0.93–0.98, O—H = 0.82 Å) and *U*_iso_(H) (in the range 1.2–1.5 times *U*_eq_ of the parent atom), after which the positions were refined with riding constraints.

### Crystal data

**Table:**

C_13_H_14_O_5_	*F*(000) = 528
*M**_r_* = 250.25	*D*_x_ = 1.390 Mg m^−^^3^
Orthorhombic, *P*2_1_2_1_2_1_	Mo *K*α radiation, λ = 0.71073 Å
Hall symbol: P 2ac 2ab	Cell parameters from 1493 reflections
*a* = 8.6170 (2) Å	θ = 5–27°
*b* = 10.4615 (3) Å	µ = 0.11 mm^−^^1^
*c* = 13.2693 (5) Å	*T* = 150 K
*V* = 1196.18 (6) Å^3^	Block, colourless
*Z* = 4	0.50 × 0.40 × 0.40 mm

### Data collection

**Table:**

Nonius KappaCCD diffractometer	1369 reflections with *I* > 2.0σ(*I*)
graphite	*R*_int_ = 0.036
ω scans	θ_max_ = 27.4°, θ_min_ = 5.1°
Absorption correction: multi-scan (*DENZO*/*SCALEPACK*; Otwinowski & Minor, 1997)	*h* = −11→11
*T*_min_ = 0.91, *T*_max_ = 0.96	*k* = −13→13
8306 measured reflections	*l* = −17→17
1547 independent reflections	

### Refinement

**Table:**

Refinement on *F*^2^	Primary atom site location: structure-invariant direct methods
Least-squares matrix: full	Hydrogen site location: inferred from neighbouring sites
*R*[*F*^2^ > 2σ(*F*^2^)] = 0.033	H-atom parameters constrained
*wR*(*F*^2^) = 0.075	Method = Modified Sheldrick *w* = 1/[σ^2^(*F*^2^) + ( 0.04*P*)^2^ + 0.33*P*] , where *P* = (max(*F*_o_^2^,0) + 2*F*_c_^2^)/3
*S* = 0.96	(Δ/σ)_max_ = 0.0003
1547 reflections	Δρ_max_ = 0.21 e Å^−^^3^
163 parameters	Δρ_min_ = −0.18 e Å^−^^3^
0 restraints	

### Fractional atomic coordinates and isotropic or equivalent isotropic displacement parameters (Å^2^)

**Table:**

	*x*	*y*	*z*	*U*_iso_*/*U*_eq_	
O1	0.20180 (16)	0.58639 (12)	0.35659 (10)	0.0315	
C2	0.0428 (2)	0.53792 (18)	0.36504 (15)	0.0308	
C3	0.0572 (2)	0.41273 (17)	0.42367 (14)	0.0290	
O4	0.07235 (15)	0.30513 (13)	0.35853 (12)	0.0348	
C5	0.2332 (2)	0.27989 (17)	0.34757 (14)	0.0282	
O6	0.30017 (15)	0.31402 (12)	0.44177 (10)	0.0292	
C7	0.2137 (2)	0.42186 (16)	0.47876 (13)	0.0271	
C8	0.2876 (2)	0.54172 (17)	0.43333 (14)	0.0284	
O9	0.41116 (16)	0.58753 (14)	0.45530 (11)	0.0382	
C10	0.2143 (3)	0.4195 (2)	0.59243 (14)	0.0403	
C11	0.2581 (2)	0.14034 (17)	0.32652 (14)	0.0278	
C12	0.3486 (2)	0.10164 (19)	0.24540 (15)	0.0314	
C13	0.3662 (2)	−0.0280 (2)	0.22427 (16)	0.0349	
C14	0.2945 (3)	−0.11716 (19)	0.28505 (15)	0.0358	
C15	0.2066 (2)	−0.07955 (18)	0.36660 (15)	0.0345	
C16	0.1874 (2)	0.04930 (19)	0.38769 (15)	0.0314	
C17	−0.0516 (2)	0.63951 (19)	0.41789 (16)	0.0359	
O18	0.02305 (17)	0.66472 (14)	0.51139 (11)	0.0385	
H21	0.0028	0.5221	0.2934	0.0368*	
H31	−0.0301	0.4024	0.4712	0.0369*	
H51	0.2780	0.3352	0.2917	0.0355*	
H101	0.3207	0.4225	0.6174	0.0626*	
H103	0.1573	0.4942	0.6146	0.0621*	
H102	0.1635	0.3404	0.6145	0.0620*	
H121	0.3978	0.1652	0.2045	0.0380*	
H131	0.4316	−0.0556	0.1653	0.0416*	
H141	0.3061	−0.2081	0.2710	0.0428*	
H151	0.1588	−0.1422	0.4102	0.0424*	
H161	0.1248	0.0765	0.4455	0.0371*	
H172	−0.0541	0.7167	0.3741	0.0456*	
H171	−0.1605	0.6071	0.4292	0.0454*	
H181	−0.0132	0.7294	0.5413	0.0598*	

### Atomic displacement parameters (Å^2^)

**Table:**

	*U*^11^	*U*^22^	*U*^33^	*U*^12^	*U*^13^	*U*^23^
O1	0.0387 (7)	0.0245 (6)	0.0313 (7)	−0.0037 (6)	0.0002 (6)	0.0025 (5)
C2	0.0337 (9)	0.0244 (9)	0.0343 (10)	0.0014 (8)	−0.0037 (8)	−0.0015 (8)
C3	0.0286 (9)	0.0233 (9)	0.0352 (10)	−0.0005 (8)	−0.0006 (8)	−0.0020 (8)
O4	0.0290 (7)	0.0240 (7)	0.0513 (9)	0.0010 (6)	−0.0099 (7)	−0.0079 (6)
C5	0.0307 (9)	0.0232 (9)	0.0306 (10)	−0.0008 (7)	−0.0024 (8)	−0.0019 (7)
O6	0.0303 (6)	0.0239 (6)	0.0335 (7)	0.0014 (6)	−0.0054 (6)	−0.0044 (5)
C7	0.0296 (9)	0.0226 (8)	0.0290 (9)	−0.0003 (8)	0.0000 (8)	−0.0009 (7)
C8	0.0335 (10)	0.0226 (8)	0.0292 (9)	−0.0013 (8)	0.0028 (8)	−0.0053 (7)
O9	0.0341 (7)	0.0322 (7)	0.0482 (8)	−0.0095 (6)	−0.0005 (7)	−0.0095 (7)
C10	0.0500 (12)	0.0428 (12)	0.0282 (10)	0.0028 (11)	0.0006 (9)	0.0014 (9)
C11	0.0312 (9)	0.0217 (8)	0.0307 (9)	−0.0016 (7)	−0.0040 (8)	−0.0008 (7)
C12	0.0320 (9)	0.0276 (9)	0.0345 (10)	−0.0016 (8)	0.0004 (8)	0.0022 (8)
C13	0.0372 (10)	0.0314 (10)	0.0360 (11)	0.0031 (9)	0.0022 (9)	−0.0041 (8)
C14	0.0418 (11)	0.0245 (9)	0.0411 (11)	0.0017 (9)	−0.0031 (10)	−0.0014 (8)
C15	0.0414 (10)	0.0251 (9)	0.0370 (10)	−0.0042 (9)	−0.0013 (9)	0.0017 (8)
C16	0.0350 (10)	0.0283 (9)	0.0308 (9)	−0.0015 (8)	0.0014 (8)	0.0013 (8)
C17	0.0382 (10)	0.0291 (10)	0.0406 (11)	0.0050 (9)	−0.0079 (10)	−0.0051 (9)
O18	0.0442 (8)	0.0326 (7)	0.0385 (8)	0.0078 (6)	−0.0058 (7)	−0.0091 (6)

### Geometric parameters (Å, °)

**Table:**

O1—C2	1.465 (2)	C10—H103	0.968
O1—C8	1.342 (2)	C10—H102	0.981
C2—C3	1.528 (3)	C11—C12	1.390 (3)
C2—C17	1.511 (3)	C11—C16	1.392 (3)
C2—H21	1.025	C12—C13	1.394 (3)
C3—O4	1.425 (2)	C12—H121	0.957
C3—C7	1.537 (3)	C13—C14	1.379 (3)
C3—H31	0.988	C13—H131	1.007
O4—C5	1.418 (2)	C14—C15	1.379 (3)
C5—O6	1.422 (2)	C14—H141	0.974
C5—C11	1.502 (2)	C15—C16	1.387 (3)
C5—H51	1.016	C15—H151	0.967
O6—C7	1.438 (2)	C16—H161	0.981
C7—C8	1.530 (2)	C17—O18	1.422 (2)
C7—C10	1.508 (2)	C17—H172	0.995
C8—O9	1.203 (2)	C17—H171	1.009
C10—H101	0.975	O18—H181	0.844
			
C2—O1—C8	109.66 (14)	C7—C10—H103	106.8
O1—C2—C3	105.05 (15)	H101—C10—H103	110.3
O1—C2—C17	107.19 (15)	C7—C10—H102	108.1
C3—C2—C17	114.22 (17)	H101—C10—H102	110.2
O1—C2—H21	107.4	H103—C10—H102	111.3
C3—C2—H21	111.2	C5—C11—C12	120.49 (16)
C17—C2—H21	111.3	C5—C11—C16	119.63 (17)
C2—C3—O4	112.06 (15)	C12—C11—C16	119.86 (17)
C2—C3—C7	105.08 (15)	C11—C12—C13	120.03 (18)
O4—C3—C7	104.91 (14)	C11—C12—H121	119.0
C2—C3—H31	110.9	C13—C12—H121	120.9
O4—C3—H31	111.8	C12—C13—C14	119.47 (19)
C7—C3—H31	111.8	C12—C13—H131	119.8
C3—O4—C5	107.37 (13)	C14—C13—H131	120.8
O4—C5—O6	105.05 (15)	C13—C14—C15	120.82 (19)
O4—C5—C11	109.85 (15)	C13—C14—H141	120.1
O6—C5—C11	110.45 (15)	C15—C14—H141	119.0
O4—C5—H51	109.9	C14—C15—C16	120.09 (18)
O6—C5—H51	110.1	C14—C15—H151	120.7
C11—C5—H51	111.3	C16—C15—H151	119.2
C5—O6—C7	106.65 (13)	C11—C16—C15	119.71 (18)
C3—C7—O6	104.08 (14)	C11—C16—H161	119.9
C3—C7—C8	103.21 (15)	C15—C16—H161	120.4
O6—C7—C8	107.03 (14)	C2—C17—O18	106.96 (16)
C3—C7—C10	118.52 (17)	C2—C17—H172	108.2
O6—C7—C10	109.08 (16)	O18—C17—H172	111.6
C8—C7—C10	113.95 (16)	C2—C17—H171	109.5
C7—C8—O1	110.80 (15)	O18—C17—H171	110.7
C7—C8—O9	126.80 (18)	H172—C17—H171	109.8
O1—C8—O9	122.17 (17)	C17—O18—H181	113.1
C7—C10—H101	110.0		

### Hydrogen-bond geometry (Å, °)

**Table:**

*D*—H···*A*	*D*—H	H···*A*	*D*···*A*	*D*—H···*A*
C10—H103···O18	0.97	2.53	3.233 (3)	130
C13—H131···O9^i^	1.01	2.58	3.289 (3)	128
C14—H141···O1^ii^	0.97	2.59	3.340 (3)	134
O18—H181···O9^iii^	0.84	2.02	2.801 (3)	153

Symmetry codes: (i) −*x*+1, *y*−1/2, −*z*+1/2; (ii) *x*, *y*−1, *z*; (iii) *x*−1/2, −*y*+3/2, −*z*+1.
